# An assessment of referrals to a liaison psychiatry team within a large district general hospital – Completing the Cycle

**DOI:** 10.1192/bjo.2021.306

**Published:** 2021-06-18

**Authors:** Ivan Shanley, Sophie Tillman, Shruti Lodhi, Shazia Shabbir

**Affiliations:** Surrey and Borders NHS Foundation Trust, Frimley Park Hospital

## Abstract

**Aims:**

In 2019 members of the Liaison Psychiatry Department at Frimley Park Hospital completed an audit of the referrals to the service1. The quality of referrals was found to be highly variable, for example only 28% included a risk assessment and frequently omitted both past psychiatric and past medical histories. As such an intervention was designed involving three parts;

Multidisciplinary education of staff

New and more readily available referral guidelines

New referral form

This re-audit seeks to complete the audit cycle and assess the impact of the intervention.

**Method:**

The first 50 referrals to the Liaison Psychiatry Department of Frimley Park Hospital during February 2021 were assessed using the following criteria:

Staff type, referral source, physically fit for assessment, physical cause ruled out, drugs / alcohol involved, appropriate reason for referral, clinical question asked, did final diagnosis match referral diagnosis, risk assessment included, information about admission included, past psychiatric history included and past medical history included.

The percentage of referrals received for each criterion (e.g. the percentage with a risk assessment completed) was then derived from the data.

**Result:**

There has been a marked improvement in a variety of areas. The percentage of referrals containing a risk assessment increased from 28% to 96%. This is likely due to the risk box now requiring an entry prior to being able to submit the referral form. Similarly the percentage containing past psychiatric history has risen from 38.8% to 90%. Previously 46.2% of referrals contained a working diagnosis which was not consistent with the clinical picture, but again this has improved, with 60% of initial diagnoses now matching the final outcome. There are however areas for improvement. Only 14% of referrals contained a specific clinical question, which is lower than the 20% achieved previously. This may be because the new referral form does not provide a specific free text box for this.

**Conclusion:**

The intervention yielded a marked improvement in the quality of referrals received by the Liaison Psychiatry Department at Frimley Park Hospital, and it is the intention to continue to use the current process. Based on the new results we will look to make small adjustments, for example adding a free text box for a specific clinical question and emphasising the importance of this information.

